# Associations between the degree of correction of hypoglycemia and ICU mortality

**DOI:** 10.1186/cc14447

**Published:** 2015-03-16

**Authors:** R Van Hooijdonk, JM Binnekade, A Abu-Hanna, F Van Braam Houckgeest, LS Hofstra, J Horn, MA Kuiper, NP Juffermans, HL Van den Oever, JP Van der Sluijs, PE Spronk, MJ Schultz

**Affiliations:** 1 Academic Medical Center, Amsterdam, the Netherlands; 2 Tergooi Hospitals, Hilversum, the Netherlands; 3 Scheper Hospital, Emmen, the Netherlands; 4 Medical Centre Leeuwarden, the Netherlands; 5 Deventer Hospital, Deventer, the Netherlands; 6 Medical Center Haaglanden, The Hague, th eNetherlands; 7 Gelre Hospitals, Apeldoorn, the Netherlands

## Introduction

It is conjectured that transition of hypoglycemia to hyperglycemia may be more harmful than hypoglycemia itself. We investigated the association between the degree of correction of hypoglycemia and ICU mortality in patients under moderately strict to strict glycemic control.

## Methods

This is a retrospective analysis from a pooled cohort from seven ICUs in the Netherlands over 6 years. ICU patients who developed hypoglycemia (<70 mg/dl) were included. We excluded patients who were readmitted, and patients with hypoglycemia in whom no follow-up blood glucose measurement was performed within 8 hours. We determined the association between three measures of correction of hypoglycemia within 8 hours after hypoglycemia and ICU mortality: predefined ranges of the 'highest blood glucose level' (<80 mg/dl; 80 to 110 mg/dl; 110 to 150 mg/dl (reference category); 150 to 180 mg/ dl; and >180 mg/dl); quartiles of the 'delta glucose', defined as the difference between minimum and maximum blood glucose level with the third quartile as reference category; and quartiles of the 'standard deviation' of the blood glucose level with the third quartile as reference category.

## Results

In total, 4,516 ICU patients developed at least one episode of hypoglycemia. In three separate multivariate analyses for each of the three measures we adjusted for the respective confounders. The category 80 to 110 mg/dl of the 'highest blood glucose level' was associated with increased mortality compared with the reference category (odds ratio (OR) = 1.31, 95% confidence interval (CI) = 1.06 to 1.61). The lowest quartile of the 'delta glucose' (OR = 1.32, 95% CI = 1.03 to 1.69) and the lowest quartile of the 'standard deviation' (OR = 1.55, 95% CI = 1.23 to 1.96) were associated with higher ICU mortality than their reference categories.

## Conclusion

Not the transition to hyperglycemia, but insufficient recovery from hypoglycemia is associated with an increased ICU mortality in patients under moderately strict or strict glucose control with insulin.

